# Supplementary material for: Complexome profiling of the Chlamydomonas psb28 mutant reveals TEF5 as an early PSII assembly factor
Source: Plant Cell. 2025 Mar 17;37(6):koaf055. doi: 10.1093/plcell/koaf055 (PMC12164590; doi:10.1093/plcell/koaf055)
Supplement: koaf055_Supplementary_Data [file koaf055_supplementary_data.zip › psb28_interactive.zip!/plots/intensity/qualityPlots/peptides/rps3_Cre-1.g2716997.t1.p1.html]

### Cre-1.g2716997.t1.p1

Trivial name: rps3  
SeqCoverage [median]: 14.60  
Mass: 81.85 kDa  
  
 

| Sequence | MissedCleavage | Mass [Da] | wtIntensity | muIntensity | Score |
| --- | --- | --- | --- | --- | --- |
| ANIDYAYR | 0 | 984.47 | 0.00 | 346.08 | 98.52 |
| ANLALTPK | 0 | 826.49 | 185.95 | 0.00 | 62.66 |
| ENPNALLQR | 0 | 1053.56 | 0.00 | 327.00 | 55.68 |
| IQNLIR | 0 | 755.47 | 0.00 | 1234.13 | 112.27 |
| LQTAFLTQIESQRK | 1 | 1661.91 | 0.00 | 899.10 | 32.07 |
| LVDDNQAMANESR | 0 | 1461.65 | 0.00 | 393.46 | 91.55 |
| SGSNLRDNLAPVK | 1 | 1369.73 | 0.00 | 580.47 | 50.15 |
| SYALINNLK | 0 | 1034.58 | 0.00 | 867.96 | 56.01 |
| TSASGTSVLK | 0 | 949.51 | 0.00 | 2063.13 | 109.11 |
| VVNNFVK | 0 | 818.47 | 0.00 | 671.82 | 85.31 |
| YLNIISK | 0 | 849.50 | 0.00 | 370.02 | 70.00 |
|  |  |  | 186.0 | 7753.2 |  |
